# Variation in male spermiation response to exogenous hormones among divergent populations of Red-eyed Treefrogs

**DOI:** 10.1186/s12958-016-0216-3

**Published:** 2016-12-05

**Authors:** Leah E. Jacobs, Jeanne M. Robertson, Kristine Kaiser

**Affiliations:** Department of Biology, California State University, Northridge, CA 91330-8303 USA

## Abstract

**Background:**

The non-lethal collection of sperm from live males is an important component for multiple captive-breeding techniques, including assisted reproductive technology (ART) protocols, sperm cryopreservation and in vitro fertilization. However, in amphibians, the type and amount of hormone necessary to induce spermiation can be highly variable, even among closely related species. We are unaware of any studies that have examined the spermiation response to exogenous hormones across highly differentiated populations within a species.

**Methods:**

We examined variation in sperm viability and production in response to the hormone LHRH among four divergent populations of the Red-eyed Treefrog (*Agalychnis callidryas*). We hypothesized that these highly differentiated populations would show variability in sperm count and viability in response to two dosages, 2 μg/g and 4 μg/g, of the hormone LHRH. We collected spermic urine 3 h post injection (PI). We then examined variation in spermiation at 3, 7, 12, and 24 h PI of LHRH for two allopatric populations that previously showed evidence of premating behavioral isolation.

**Results:**

One population of Red-eyed Treefrog exhibited reduced sperm viability but not count in response to the hormone LHRH compared to all other populations. In addition, we found peak viability at 3 h PI for the allopatric population comparison. There was no difference in sperm production within or between populations at 3, 7, 12, or 24 h PI. For both studies, intrapopulation variation was high.

**Conclusion:**

ART often focuses on threatened species with small, isolated populations, which could evolve localized differences due to the evolutionary process of drift and selection. The high variation in response and the population-level differences in sperm viability we observed demonstrate that practitioners of ART should consider the possibility of divergent responses to hormones which may affect study design and animal receptivity to ART protocols.

**Electronic supplementary material:**

The online version of this article (doi:10.1186/s12958-016-0216-3) contains supplementary material, which is available to authorized users.

## Background

Amphibians are facing extinction rates that exceed those of birds, mammals, or reptiles [1]. Since 1980, at least 165 species of amphibians have gone extinct and approximately 30% of the amphibians assessed by the International Union for Conservation of Nature (IUCN) are currently threatened with extinction [1–3]. Assisted reproductive technologies (ART) have been increasingly implemented in captive-breeding settings to augment amphibian populations. Many ART protocols for amphibians combine traditional breeding with laboratory methods (e.g., the use of hormones) for successful breeding and induction of viable oocytes and sperm for cryopreservation and/or in vitro fertilization [4, 5]. Historically, ART protocols used sperm that were collected from testes macerates, thus sacrificing males [6–8], however, within the past 20 years, exogenous hormones have been used to obtain sperm non-invasively [9]. To date, at least 12 species of anurans representing seven families have been bred using ART [10].

One of the most common and effective hormones used in amphibian reproductive technologies is gonadotropin-releasing hormone (GnRH, known in the ART literature as luteinizing-hormone releasing hormone [LHRH]; [11]). Although LHRH has proven useful in ART in anurans, the dose of hormone required to induce spermiation or ovulation and the latency to spermiation varies by orders of magnitude among frog families [10]. For example, a dosage of 0.28–0.58 μg/g LHRH is sufficient to promote spermiation in leptodactylids [12], whereas a 2 μg/g LHRH dose is more effective for myobatrachids [13]. Further, the dose for closely related species within a genus can vary [9], as can the optimal time post-hormone administration for collection of gametes [10].

Currently, hormonal ART protocols are generally developed and optimized for species, not populations, regardless of how genetically or geographically isolated the source populations may be. However, population-level differences may result in variable responses to exogenous hormones that may mask or limit the efficacy of a protocol. To our knowledge, there has been no attempt to understand whether the response to hormones varies among populations within a species. We thus assessed the spermiation response to LHRH in highly divergent intraspecific populations of Red-eyed Treefrogs (*Agalychnis callidryas*).

The Red-eyed Treefrog is a broadly distributed Neotropical phyllomedusine frog [14]. This species exhibits genetic and phenotypic divergence across populations [15–17], including strong differentiation in color pattern and body size [17], skin antimicrobial peptides [18], and advertisement calls (unpubl. data). Allopatric populations show assortative mating for local males, indicating that some premating behavioral reproductive isolation has evolved [19]. In addition, offspring of individuals from parapatric or allopatric crosses have lower fitness than parental crosses (unpubl. data). However, genetic analyses using nuclear and mitochondrial DNA indicate that these populations represent a single intraspecific lineage [15, 16].

Red-eyed Treefrogs are well suited for testing the hypothesis that the extensive phenotypic and genetic divergence among populations is associated with population-level physiological differences. We used exogenous LHRH to test the effects of hormone dosage and time post-injection on sperm viability and count. We predicted that if reproductive physiology has shifted with other divergent traits, ejaculate characteristics would vary among populations and over time.

## Methods

### Animal collection, and housing

**Fig. 1:**
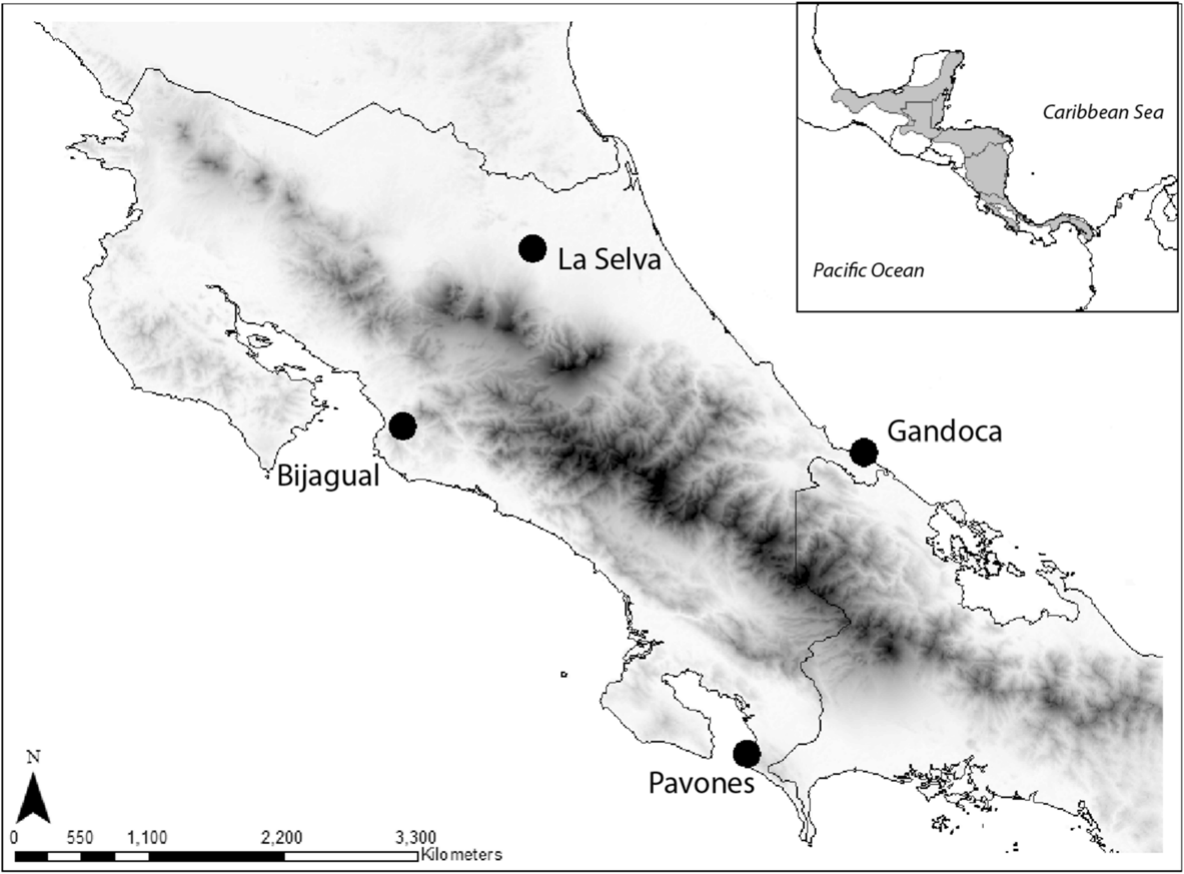
Four geographically isolated populations near the southern end of the range of *A. callidryas*. Bijagual (9.5188, -84.3774) and Pavones (8.4204, -83.1069) are allopatric to La Selva (10.4327, -84.0080) and Gandoca (9.6332, -82.6556), separated by the Talamancan mountain range (shown in *black*). ASTER Costa Rica image was retrieved from https://lpdaac.usgs.gov, maintained by the NASA EOSDIS Land Processes Distributed Active Archive Center. The data product for the image was provided by NASA. Inset: The geographic range of *A. callidryas* in Central America

Adult frogs were collected from Costa Rica in the summer of 2014 and transported to the California State University, Northridge (CSUN) vivarium. To ensure that all males were reproductively mature and receptive collected all individuals in a natural breeding aggregation. Animals were housed at a constant temperature of 26.7 ± 3.0 °C and under a 12:12 h photocycle; this temperature and light cycle reflects the natural environmental conditions of their breeding season. We collected males from each of four populations that vary in the extent of genetic and phenotypic differentiation across their range (Fig. 1). We housed frogs by population, grouping up to four individuals per each screen-topped glass aquarium (50.8 × 27.9 × 33.0 cm). Tanks were lined with 2 cm of pea gravel (Silver Rock Shallow Creek Aquarium Gravel, San Antonio, Texas), and included a plastic plant (Petco Araceae Terrarium Plant Reptile Décor, San Antonio, Texas), water dish (77.0 ml), and a fogger system (ZooMed ReptiFogger, San Luis Obispo, CA) which was run continuously, providing a mean relative humidity of 80%. We rinsed the gravel and fed the frogs to satiation twice weekly with 2-weeks old crickets (*Acheta domestica*; Flukers, Port Allen, LA) gut loaded with calcium.

### Hormone administration

We diluted hormone (LHRHa; Des-GLY10, D-ALA6)-LH-RH ethylamide ACE, an analog of luteinizing hormone-release hormone, Sigma Aldrich, Milwaukee, WI) in simplified amphibian Ringer’s solution (SAR; 113 mM NaCL, 2 mM KCl, 1.35 mM CaCl_2_, 1.2 mM NaHCO_3_) to create a 0.8 mg/ml dilution of hormone to SAR. Immediately prior to injections, we weighed each frog and administered hormone adjusted for each animal’s mass (see below for explanation of dosages; [11]). The hormone was administered in 100 μl of SAR with a 1-cc insulin syringe via a ventral intraperitoneal injection. After each injection, we isolated males in individual cages (29.8 × 20.3 × 19.7 cm; PetCo Pet Keeper, San Antonio, Texas), which were covered with wet paper towels to maintain humidity until spermiation. Research on Fowler Toads (*Anaxyrus fowleri*) showed that subsequent injections every 7 d did not adversely affect sperm count over a breeding season [5]. Thus, we allowed 1–4 weeks between injections. We also controlled for seasonal effects by randomizing the treatment order for each individual in each experiment and by conducting trials during the natural breeding season for *A. callidryas* (May through November; [20]). Through all experiments, we attempted to minimize handling stress by 1) allowing only experienced handlers to work with frogs; 2) handling animals only during injection and spermiation; 3) spermiating frogs only once per injection and 4) returning animals to holding tanks immediately after procedures.

### Dose-dependence trials

To test for population-level differences in hormone dose-dependence on spermiation, we injected males (*n* = 5) from each of the four populations. All males in this study received each of the three dose treatments (0, 2, or 4 μg/g LHRHa) in a randomized order*.* Preliminary trials indicated that hormone doses ranging from 0.1 to 1 μg/g LHRH were insufficient to elicit spermiation in Red-eyed Treefrogs (Jacobs; unpubl. data). We collected spermic urine samples, as described below, from each male 3 h (±10 min) post administration of hormone [5].

### Time-dependence trials

To test whether highly differentiated populations differ in spermiation responses over time, we injected males from two allopatric populations that exhibit partial premating reproductive isolation: La Selva (*n* = 7) and Bijagual (*n* = 10). Due to limited number of individuals some males in the dose-dependence trials were also used in the time-dependence trials (La Selva (*n* = 5); Bijagual (*n* = 2)). We used a dose of 4 μg/g LHRH for all injections because we measured similar sperm concentrations in response to 4 μg/g LHRH from both populations in previous trials (see Results; dose-dependence trials; Additional file 1). Each male in this study was injected a total of four times: once for each treatment (spermiation at 3, 7, 12, and 24 h post injection) on a randomized schedule over a period of 4 weeks.

### Sperm count and viability

We collected spermic urine for sperm count and viability estimates by firmly grasping each frog by the front legs and holding it over a Petri dish until it urinated (usually 5–10 s). We collected the sample from the Petri dish using a sterile 100 μl pipette and used approximately half the sample for sperm count analyses and placed the remaining sample in a 1.5 ml microcentrifuge tube for sperm-viability analysis.

Sperm count was conducted using a hemocytometer. We counted each sample twice and used the mean in analyses. Our measures were highly repeatable (repeatability SPSS 20.0; *r* = 0.95; [21]). To determine sperm viability (live sperm/total sperm counted), we used SYBR-14 and propidium iodide, a staining method commonly used for amphibians (e.g., [10, 13, 22, 23]). Briefly, we homogenized samples with 5 μl of a 1:50 dilution of SYBR-14 (Invitrogen L-7011, Canoga Park, CA) and incubated for 7 min; we then incubated with 2 μl propidium iodide for 7 min. Both incubations were done in the dark. We analyzed stained samples using fluorescent microscopy under 100× magnification (Zeiss inverted fluorescent microscope, 2012, Dublin, CA). We counted no fewer than 20 sperm per sample (baseline number for examining viability of sperm in amphibians; [24]); samples with fewer than 20 sperm were not included in viability analyses.

### Statistical analyses

We used an ANCOVA for counts for both dose-dependence and time-dependence trials to account for body mass as a covariate and distinguish the potential role of body size on sperm production. We conducted two-way repeated measures ANOVAs on sperm viability for both dose-dependence and time-dependence trials. We used a Tukey’s post-hoc comparison of means on factors that were significant in either study. All analyses were conducted in StataIC (v. 10.1). Results were considered significant when *P* was < 0.05.

## Results

### Dose-dependence trials

None of the males produced sperm following the 0 μg/g LHRH control injections. In response to 2 μg/g LHRH, all males from Bijagual and Gandoca produced sperm; 60% (3/5) of La Selva males produced sperm and 80% (4/5) of Pavones males produced sperm. All males in the study produced sperm after an injection of 4 μg/g LHRH with the exception of one Bijagual male.

### Sperm count

There was no difference in sperm count in response to LHRH dosage (2 vs. 4 μg/g) among populations (two-way RM ANCOVA; *F*_3,45_ = 1.32, *P* = 0.30) or within (*F*_1,45_ = 0.22, *P* = 0.64) populations (Additional file 1). Body weight was not significant so was dropped from the model and counts were re-run as a two-way repeated measures ANOVA. The results did not change with body weight dropped from the model: we detected no difference in sperm count in response to either dose of LHRH among (two-way RM ANOVA; *F*_*3,45*_ *=* 0.57, *P* = 0.64) or within (*F*_*3,45*_ = 0.55, *P* = 0.46) populations.

### Viability

**Fig. 2:**
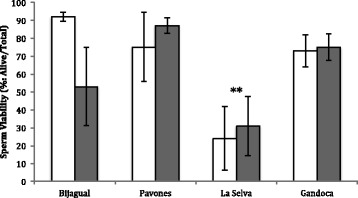
Mean (± SE) sperm viability differed among populations (*n* = 5 for all) of *Agalychnis callidryas* in response to 2 μg/g (*white bars*) or 4 μg/g (*dark gray bars*) LHRH. The La Selva population differed from other populations in sperm viability in response to the hormone LHRH (*P* < 0.001). All populations showed similar sperm viability in response to 2 vs. 4 μg/g LHRH

The viability of sperm in response to LHRH differed among populations (two-way RM ANOVA; *F*_3,41_ = 6.78, *P* < 0.01). A posthoc Tukey Kramer test showed that sperm viability in La Selva frogs was significantly lower than Bijagual frogs in response to the hormone LHRH (*P* < 0.05; Fig. 2). We found no difference in overall viability response to dosage of hormone LHRH (2 vs. 4 μg/g) (*F*_3,63_ = 0.83, *P* = 0.37) (Fig. 2).

### Time-dependence trials

We injected 10 Bijagual males with 4 μg/g LHRH for time-dependence trials; 90% (9/10) produced sperm after 3 h; 80% (8/10) after 7 and 12 h, and 70% (7/10) after 24 h. We injected 7 La Selva males; 57% (4/7) produced sperm after 3 h; 85% (6/7) after 7 h; 57% (4/7) after 12 h, and 71% (5/7) after 24 h.

### Sperm count

Body weight had no effect on sperm-count production (*F*_1,67_ = 0.05, *P* = 0.83) and was dropped from subsequent models. Sperm count did not significantly differ over time within populations (*F*_3*,68*_ = 1.58, *P* = 0.20) and no significant differences could be detected between La Selva and Bijagual in these trials (*F*_1,68_ = 1.86, *P* = 0.19; Additional file 2).

### Viability

**Fig. 3:**
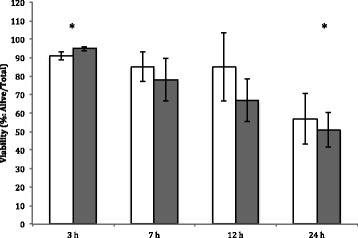
Mean (± SE) sperm viability of *Agalychnis callidryas* varied over time for both populations, Bijagual (*white bars*) and La Selva (*dark gray bars*) to 4 μg/g LHRH with the highest concentration occurring at 3 h PI and decreasing at 24 h PI (*P* < 0.05)

The interaction of population by time was not significant and was dropped from the model. Sperm viability did not differ among populations (*F*_1,48_ = 0.02, *P* = 0.90) but did differ between time points (*F*_3,48_ = 4.23, *P* = 0.013; Fig. 3). Populations had peak viability at 3 h PI and a posthoc Tukey Kramer test showed that this timepoint differed from 24 h PI (*P* < 0.05).

## Discussion

We found that exogenous hormone LHRH was effective in producing viable sperm from all populations of Red-eyed Treefrogs in our study. Collectively, males produced sperm in response to the hormone LHRH in 100% of all dose trials. For successful in vitro fertilization, a concentration of 1 × 10^5^ motile sperm/ml is needed to fertilize a clutch of 100–200 eggs [5]. Female Red-eyed Treefrogs in laboratory settings produce 50 to 200 eggs per clutch (unpubl. data). Therefore, all populations in this study produced sperm count well above this baseline, and most produced over 70% viable sperm (Fig. 2).

We detected variation among populations in sperm viability in response to LHRH. Sperm produced by La Selva frogs showed reduced viability compared to all other populations. In addition, more La Selva individuals failed to produce spermic urine than individuals from any other population: 33% (2/6) of La Selva males injected with 2 μg/g LHRH in dose-dependence trials, and 57% (4/7) of males in time-dependence trials failed to produce any sperm after 3 h, although the differences among populations were not significant (cases of non-production of spermic urine: Bijagual: 0.17% (1/6) in response to 4 μg/g, Pavones: 0.20% (1/5) in response to 2 μg/g, Gandoca: 0% (0/6) in response to 2 or 4 μg/g).

Individual and population-level responses to LHRH differed between the two experiments. For example, Bijagual produced higher sperm count in time trials relative to dose-dependence trials, while the La Selva population showed the opposite pattern. Two males per population demonstrated vastly different sperm viability between the dose-dependence trials and the time-dependence trials: for example, one Bijagual male produced 0.7% viable sperm in one trial and 90% viable sperm in a later trial at the same dosage and time point (4 μg/g and 3 h PI). High within-population variability in ejaculate characteristics has been reported across taxa [25–27]. Within amphibians, individual variation in sperm count in response to injections of LHRH was observed to be 44-fold within a population of Peron’s Treefrog (*Litoria peronii*) [22]. Fewer studies have investigated among-population variation in sperm. Hettyey and Roberts [28] found that sperm quality (longevity and motility), size, and sperm concentration collected from testis macerates varied within and among non-divergent breeding populations of *Crinia georgiana*. We are not aware of any other studies investigating interpopulation variation in sperm traits in isolated and potentially divergent populations and/or in response to exogenous hormones. More research is thus called for to reveal how population-level differences may impact the development of ART protocols for rare or threatened species that occur in isolated populations.

As with many ART studies, several caveats apply to our work. Our sample sizes were low due to permit limitations in the number of animals we were able to collect. However, despite this constraint, we detected significant differences in sperm viability in our dose-dependence trials and a significant reduction in viability over a 24 h period in our time-dependence trials. While we used the same males in both experiments, we consider this unlikely to have affected our results based on previous work with amphibians [11] and based on the fact that sperm count did not drop over the course of the experiment; indeed, one population demonstrated an increase in average sperm viability between experiments. In addition, studies on toads (*Bufo fowlerii, Bufo americanus, & Bufo valliceps*) demonstrate that repeated injections continued to be effective with no obvious declines in sperm number, viability or motility [5, 24].

We did not test for sperm quality among populations, nor did we examine genetic compatibility in an in vitro fertilization setting, important factors in validating ART protocols. For example, within Bufonidae, *Anaxyrus americanus* produced viable sperm of good quality in response to the hormone hCG. While a closely related and rare species, *Anaxyrus baxteri*, produced similar sperm counts and viability estimates in response to the same dosage of hCG, sperm also had abnormal heads [12]. In addition, in vitro studies using isolated populations of *Pseudophryne bibronii* showed evidence of high embryo mortality in outcrossed clutches indicating genetic incompatibility among populations [22]. Examining genetic compatibility, sperm quality and in vitro fertilization success are the next steps toward a conservation management plan that involves ART.

## Conclusion

To our knowledge, this study presents the first examination of hormone efficacy at inducing spermiation among highly divergent populations of an anuran. The hormone LHRH was effective at inducing spermiation and we recommend for baseline ART protocols a dosage range of 2–4 μg/g LHRH at a time point of 3 h post administration in this species, which may have broader applications for other phyllomedusines. We detected variation both within and among genetically and phenotypically divergent populations.

Documenting temporal variation, peak sperm production and viability in response to hormones are critical components of ART protocols such as cryopreservation of gametes or in vitro fertilization techniques. Sperm count and viability are highly variable over time among species and families in response to hormones [22] and one study found high intraspecific variation among populations that did not show genetic or phenotypic divergence across their range [28]. Taken together, these findings indicate that an intraspecific lineage is not enough to justify the assumption that a single ART protocol fits all, but rather that protocols should be viewed as a starting point if populations are highly differentiated and/or isolated. In addition, many ART protocols use non-threatened, closely related, species as a proxy and then test on rare species, but because rare species are often represented by small, isolated populations that may possess unique genotypes, protocol optimization should be adapted to consider and document differences in source populations [12, 23]. ART has been successfully applied to mammals, cephalopods, reptiles, birds and amphibians [5, 29–33]. Thus, these findings inform conservation and breeding management of threatened or isolated populations spanning a range of diverse taxa.

## Additional files

Additional file 1: Average sperm concentration by males, grouped by population, in response to 2 vs. 4 ug/g LHRH. No population males produced sperm after the control injection of 100 uL SARS. Sperm count was calculated as number of sperm × 10^3^ in one mL of spermic urine. (PDF 14 kb)
Additional file 2: Average sperm count by males, grouped by time. Sperm was analyzed post administration (PA), in response to 4 ug/g LHRH. Sperm count was recorded for all samples ≥20 sperm per sample and was calculated as number of sperm × 10^3^ in one mL of spermic urine. (PDF 15 kb)

## Abbreviations

ART: Assisted reproductive technology
GnRH: Gonadotropin-release hormone
hCG: Human chorionic gonadotropin
IUCN: International Union for Conservation of Nature
LHRH: Luteinizing hormone-release hormone
