# Supplementary material for: Variation in male spermiation response to exogenous hormones among divergent populations of Red-eyed Treefrogs
Source: Reprod Biol Endocrinol. 2016 Dec 5;14:83. doi: 10.1186/s12958-016-0216-3 (PMC5139111; doi:10.1186/s12958-016-0216-3)
Supplement: Additional file 1: — Average sperm concentration by males, grouped by population, in response to 2 vs. 4 ug/g LHRH. No population males produced sperm after the control injection of 100 uL SARS. Sperm count was calculated as number of sperm × 103 in one mL of spermic urine. (PDF 14 kb) [file 12958_2016_216_MOESM1_ESM.pdf]

Additional file 1

	Bijagual	Pavones	Gandoca	La Selva
Treatment	Sperm Count (x10^3)	Sperm Count (x10^3)	Sperm Count (x10^3)	Sperm Count (x10^3)
2 ug/g	89.3 ± 33.9	14.2 ± 7.36	52.6 ± 16.7	26.1 ± 15.7
4 ug/g	56.6 ± 34.0	55.9 ± 42.6	61.2 ± 23.5	61.3 ± 33.4
