# Supplementary material for: Variation in male spermiation response to exogenous hormones among divergent populations of Red-eyed Treefrogs
Source: Reprod Biol Endocrinol. 2016 Dec 5;14:83. doi: 10.1186/s12958-016-0216-3 (PMC5139111; doi:10.1186/s12958-016-0216-3)
Supplement: Additional file 2: — Average sperm count by males, grouped by time. Sperm was analyzed post administration (PA), in response to 4 ug/g LHRH. Sperm count was recorded for all samples ≥20 sperm per sample and was calculated as number of sperm × 103 in one mL of spermic urine. (PDF 15 kb) [file 12958_2016_216_MOESM2_ESM.pdf]

Additional file 2

	Hours Post Administration			
	3	7	12	24
Treatment	Sperm Count ($x10^3$)	Sperm Count ($x10^3$)	Sperm Count ($x10^3$)	Sperm Count ($x10^3$)
Bijagual	70.9 ± 24.1	35.1 ± 11.8	42.95 ± 31.6	6.3 ± 2.76
La Selva	24.28 ± 19.1	19.7 ± 10.6	10.2 ± 6.41	17.3 ± 7.58
